# Micro-CT data of early physiological cancellous bone formation in the lumbar spine of female C57BL/6 mice

**DOI:** 10.1038/s41597-021-00913-y

**Published:** 2021-05-14

**Authors:** Michael Zenzes, Paul Zaslansky

**Affiliations:** 1 Julius Wolff Institute, Charité-Universitätsmedizin Berlin, Augustenburger Platz 1, 13353 Berlin, Germany; 2 Department of Preventive and Restorative Dentistry, Charité-Universitätsmedizin Berlin, Assmanshauser Str. 4-6, 14197 Berlin, Germany

**Keywords:** Biomineralization, Bone development, Bone

## Abstract

Micro-CT provides critical data for musculoskeletal research, yielding three-dimensional datasets containing distributions of mineral density. Using high-resolution scans, we quantified changes in the fine architecture of bone in the spine of young mice. This data is made available as a reference to physiological cancellous bone growth. The scans (n = 19) depict the extensive structural changes typical for female C57BL/6 mice pups, aged 1-, 3-, 7-, 10- and 14-days post-partum, as they attain the mature geometry. We reveal the micro-morphology down to individual trabeculae in the spine that follow phases of mineral-tissue rearrangement in the growing lumbar vertebra on a micrometer length scale. Phantom data is provided to facilitate mineral density calibration. Conventional histomorphometry matched with our micro-CT data on selected samples confirms the validity and accuracy of our 3D scans. The data may thus serve as a reference for modeling normal bone growth and can be used to benchmark other experiments assessing the effects of biomaterials, tissue growth, healing, and regeneration.

## Abstract (summary)

**Table:** 

Measurement(s)	bone growth • bone mineralization involved in bone maturation
Technology Type(s)	micro-computed tomography
Factor Type(s)	age
Sample Characteristic - Organism	Mus musculus
Sample Characteristic - Environment	biological_process

Machine-accessible metadata file describing the reported data: 10.6084/m9.figshare.14062073

## Background & Summary

**Fig. 1:**
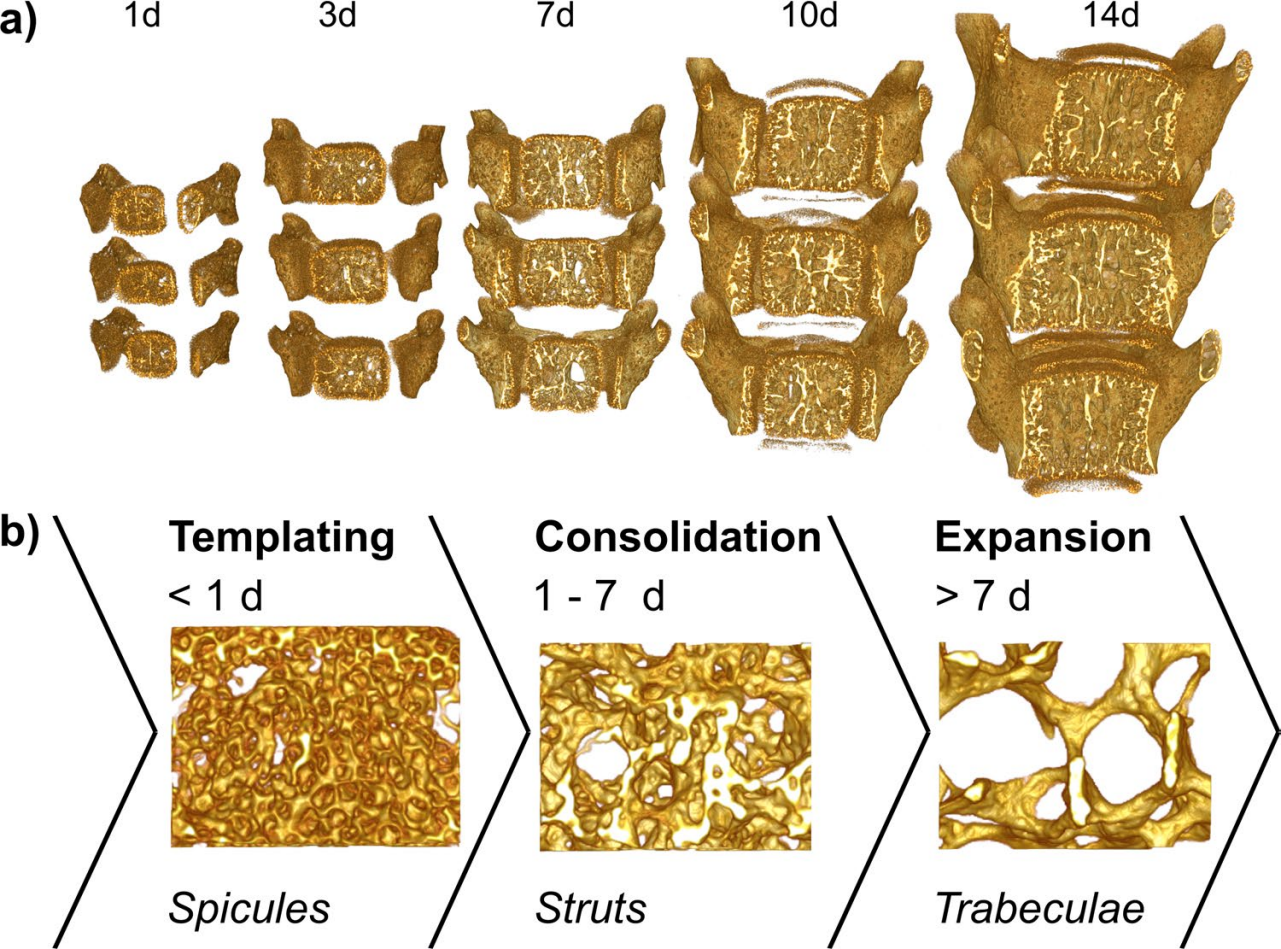
Architectural changes during physiological cancellous bone formation in the lumbar spine. (**a**) Representative 3D renderings of lumbar spine segment L3-L5, highlighting the macroscopic growth as well as the exposed cancellous bone network inside the vertebral bodies. The segments increase in size and develop anatomical features, leading to the emergence of the mature geometry within 2 weeks. (**b**) 3D views of samples of identical volume (410 × 310 × 320 µm^3^) within the vertebral body at 1-, 3-, and 7 days. These reveal three distinct phases of mineralized architecture formation: templating, consolidation, and expansion^1^.

The spine is a central skeletal element in all mammals, essential for locomotion. The vertebral bodies of the spine comprise cancellous bone that changes radically during growth. In mice, these developments take place within the first 14 days of life (Fig. 1). Growth is accompanied by the maturation of the mineralized material affecting both mineral density and bone architecture^1^. Physiological growth in mice entails a swift spatial and temporal rearrangement of the mineralized material in these bones: a process partially recapitulated during healing and regeneration^1–3^.

The early stages of cancellous bone formation cannot be studied in humans, requiring reliable animal models. Murine skeletal models have become important adjuncts to musculoskeletal research in humans, widely used in bone biomechanical studies^1–3^. Despite the widespread use of this model, only sparse data is available publicly describing spine development in 3D. In particular, there is to date an almost total lack of data representing physiological cancellous bone modeling after birth.

3D imaging has become the gold-standard when investigating mineralized tissues^1,3–5^. The acquisition of micro-CT data requires dedicated imaging infrastructure, considerable computation power, data processing, and storage, building on substantial expertise and requiring significant time to procure and process. The first data processing step entails the reconstruction of a large number of 2D radiographs obtained from different perspectives of the sample into a series of virtual cross-sectional slices that serve as a basis for 3D image analysis and simulation.

Our observer-independent imaging and analysis approach of the micro-CT data quantifies the rate and extent of the transition of cancellous bone architecture in 3D. Within 14 days the mineralized lumbar spine undergoes extensive transitions from an immature, spongy template evolving into highly oriented cancellous bone. Three phases of physiological bone formation are observed (Fig. 1b): Initially, templating yields foamy, mineralized spicules. During consolidation in the first 7 days after birth, spicules condense into struts and form primitive trabeculae. The higher bone volume ratio (BV/TV) shifts to a lower BV/TV, suggesting that the deposited mineralized tissue is a repository of bone mineral used in later developmental stages. The trabeculae then expand, increase in mineral density, into a preferentially cranial-caudal, lattice-like trabecular bone configuration. Swift growth ensues such that by day 14, the young lumbar spine exhibits all features observed in the mature animal.

The present high-resolution (2.5 µm/pixel) data provide a series of longitudinal snapshots of cancellous bone evolution in normal research-grade, prepubertal, female C57BL/6 mice^6^. By focusing on a homogenous study population, we eliminated the possible effects of sex hormones and different genetic backgrounds. While other studies focused on aging or cancellous bone degeneration, the current data depict physiological growth within the first two weeks post-partum^1,7,8^. Importantly, the high-resolution is key for resolving and tracking details in the micrometer-sized mineralized struts and spicules^1^. This micro-CT data is therefore provided together with phantom scans of defined densities (0.25 g/cc and 0.75 g/cc mineral) intended to be used for mineral density calibration. Such calibration makes it possible to quantify the changes in mineral density that accompanies bone growth and modeling^1^.

Our datasets provide a previously unavailable reference for normal cancellous bone growth at a very young age in 3D, not easily accesible in very high detail (Table 1). The scans can be used for comparison with genetically modified mouse strains, to create 3D models for numerical simulations, or as a blueprint for substrates made of biomaterials or implants which could be employed in healing and regeneration studies^1^. By sharing our datasets, in line with animal welfare ethics concepts of 3R^9^, we potentially reduce the number of animals required as reference/control for studies quantifying normal bone phenotypes. We further suggest that the datasets are essential for both better understanding pathological tissue growth and to gain insights into the factors that may influence early bone architecture formation. Changes in bone architecture are prime identifiers of both health and disease states.

**Table 1:** Overview of datasets.

Label	Age {days}	Sex	Scan segments	Images	Size	Format	Weight {g}
P01–05	1	female	L5-L3	1017	534 MB	8-bit png	1.51
P01–06			L6-L3	1282	734 MB	8-bit png	1.043
P01–07			L5-L3	1106	583 MB	8-bit png	1.199
P01–08			L5-L3	1265	727 MB	8-bit png	1.43
P03–07	3		L5-L3	1292	827 MB	8-bit png	2.389
P03–14			L5-L3	1195	616 MB	8-bit png	1.74
P03–15			L5-L3	1150	826 MB	8-bit png	1.872
P07–03	7		L5-L2	1646	2.4 GB	8-bit png	4.69
P07-04			L5-L3	1568	1.7 GB	8-bit png	4.722
P07–10			L5-L3	1416	1.5 GB	8-bit png	3.522
P07–12			L5-L3	1534	1.3 GB	8-bit png	4.04
P10-04	10		L5-L3	1882	2.6 GB	8-bit png	5.472
P10-05			L5-L3	1794	2.4 GB	8-bit png	6.531
P10-06			L5-L3	1794	2.1 GB	8-bit png	5.963
P10-08			L5-L3	1932	2.4 GB	8-bit png	7.003
P14-02	14		L5-L3	2271	3.2 GB	8-bit png	9.618
P14-06			L5-L3	2108	2.8 GB	8-bit png	7.767
P14-08			L5-L3	2308	4.3 GB	8-bit png	9.067
P14-09			L5-L2	2013	2.8 GB	8-bit png	8.372
Cali25				1254	286 MB	8-bit png	
Cali75				1254	339 MB	8-bit png	

The 21 datasets contain 19 datasets of female C57BL/6 mice between 1–14 days post-partum as well as 2 scans of phantoms of defined densities. The lumbar spine segments L3-L5 are included in all datasets. In some cases, neighboring vertebral bodies were included in the sub-scans. The datasets contain 1000–2300 8-bit png images resulting in a file size of 500MB-4GB. The animal (whole-body) weight is provided in grams.

## Methods

**Fig. 2:**
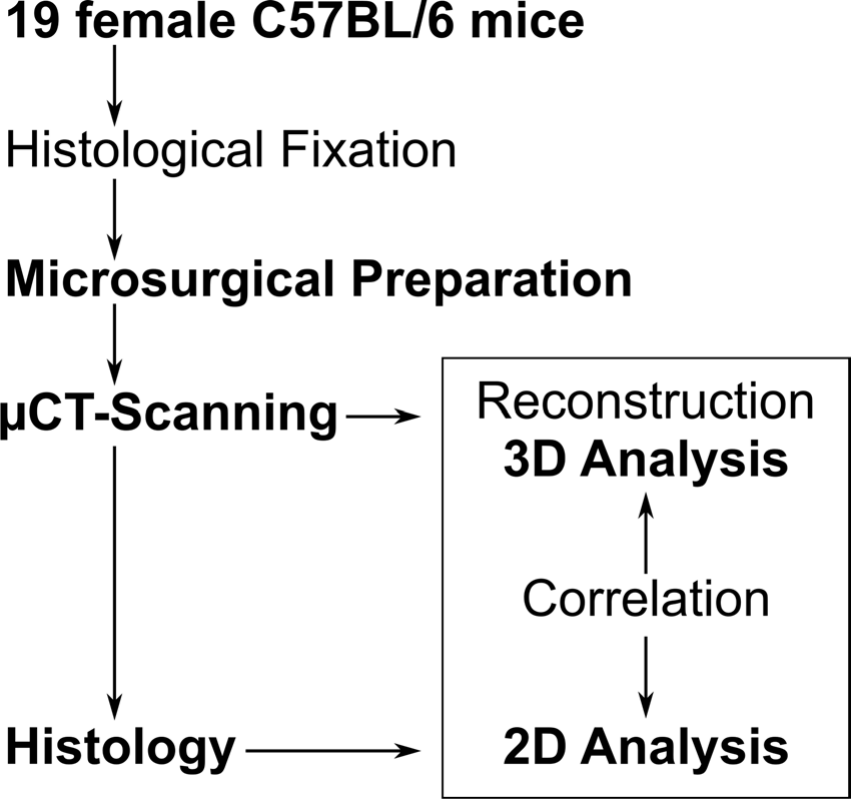
Flowchart of Materials and Methods. 19 mice underwent histological-fixation before they were prepared microsurgically. The extracted lumbar spine segments were all stored in EtOH until micro-CT scanning. The data were then reconstructed and analyzed. The samples were embedded (Technovit 9100, Kulzer GmbH, Hanau, Germany) and slices were stained and analyzed using conventional 2D histomorphometry. The results were analyzed for correlation (Fig. 3) between the micro-CT and histological slices^1^.

Lumbar spine segments from 19 female C57BL/6 mice 1, 3, 7, 10, and 14 days of age (n = 4 for 1, 7, 10, 14 days; n = 3 for 3 days) were extracted micro-surgically (Fig. 2). All animals were obtained from healthy litters at the Max Planck Institute of Molecular Genetics, Berlin, Germany. The animals were not included in any animal experiments: they were discarded from a previous study of normal skeletogenesis following CO_2_-euthanasia, and  stored at −80 °C^4^. Under the German “Tierschutzgesetz” (Landesamt für Gesundheit und Soziales, LaGeSo Berlin, Germany), no ethical approval is therefore required. The body weights of the 19 animals are listed in Table 1. For spine removal, the frozen carcasses were thawed at 4 °C for 24 hours in fixation medium (4% PFA / 70% EtOH). During dissection, each animal was gently fixated on a polystyrene board with hind- and forelimbs extended, to facilitate excision while minimizing strain on the soft tissue. Microsurgery was performed under a stereomicroscope and a cold light source. Throughout the entire procedure, the samples were repeatedly irrigated. Whole spine segments (vertebral bodies L1–L6) were extracted and stored in 70% EtOH at 4 °C until scanning.

**Fig. 3:**
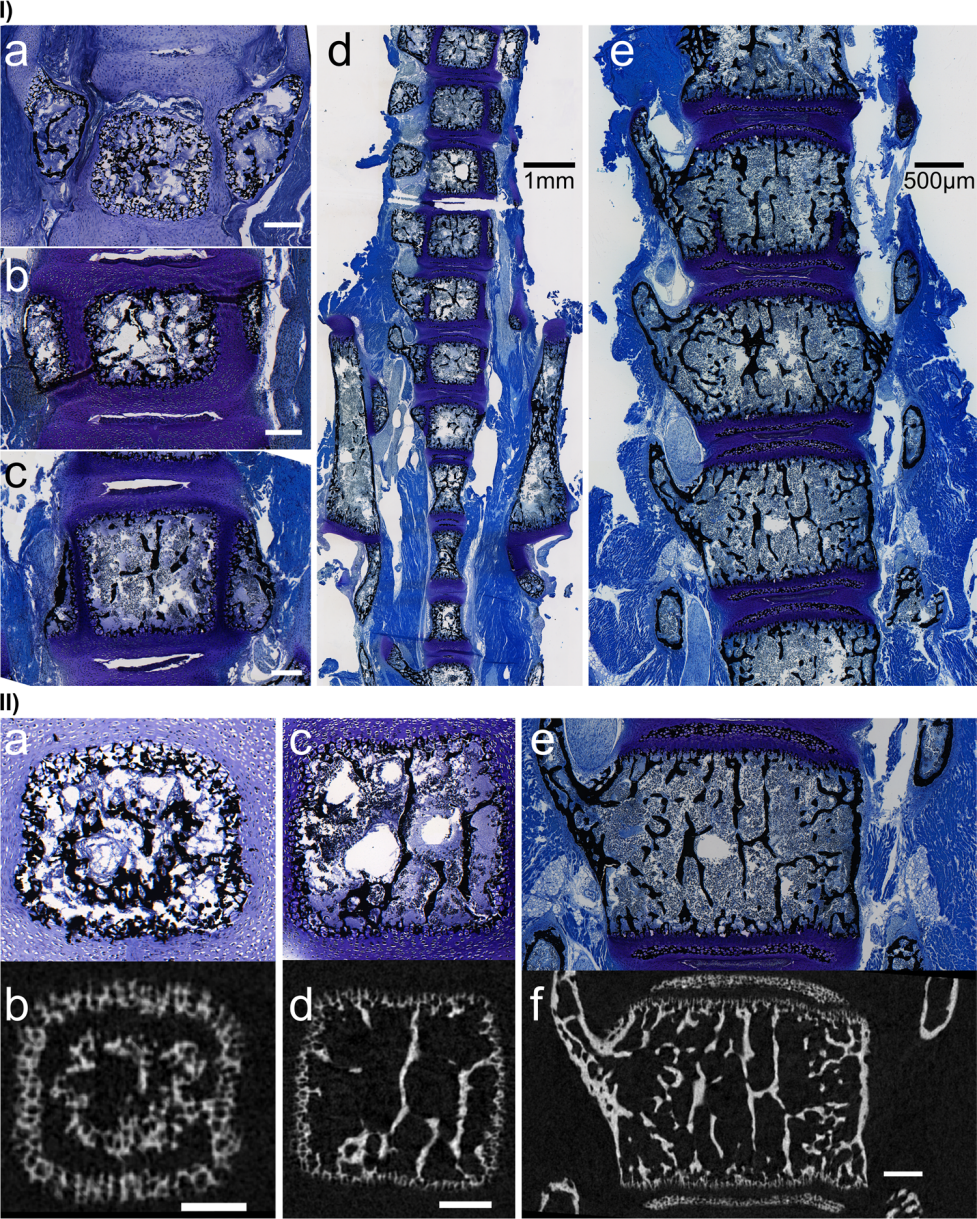
(**I**) Von Kossa - Toluidine Blue staining from 1–14 days (compare Fig. 1a). Mineralized material (black); Chondrogenic tissue (metachromatic shades of blue). Scale bar a–c = 200 µm. (**a**) The 1-day-old vertebral body is a delicate network of mineralized spicules, surrounded by a layer of chondrogenic cells. The vertebral arches are pre-shaped in cartilaginous tissue. (**b**) At 3 days, the vertebral body shows signs of mineral material condensation in its center. It is surrounded by an abundance of chondrogenic cells. (**c**) The 7-day-old vertebral body continues to consolidate the mineralized spicules into struts and early trabeculae. The outer chondrogenic surface is thinning. (**d**) Histological overview of the excised lumbar spine, sacral spine, and hip bones highlighting the anatomic complexity of this region at 10 days of age. The general trends observed continue and the vertebral bodies grow. (**e**) At 14 days a nearly mature structure, with fused vertebral body and arches, can be observed. Von Kossa stains the mineralized epiphyseal ‘caps’ between the vertebral body and the intervertebral discs in black. The growth zones are reduced to the cranial and caudal surface of the vertebral body. (**II**) (reprinted with permission granted by Elsevier^1^): Micro-CT matched with Histology. Histological sections (a,c,e) match with 2D virtual slices in the micro-CT (b,d,f) as seen in longitudinal sections of vertebral bodies aged 1 day (a,b), 7 days (c,d), and 14 days (e,f). The von Kossa/Toluidine blue staining shows mineralized material in black, cartilage, and soft tissue in metachromatic shades of blue. In the micro-CT images, only mineralized material is visible, represented by shades of grey. Scale bar = 200 µm^1^.

The samples were scanned in a Skyscan 1172 benchtop micro-CT (Bruker Micro-CT, Kontich, Belgium). A custom-made specimen holder was centered on the rotating stage, containing a polyethylene tube with each sample in an EtOH atmosphere (placed on ethanol saturated foam). The samples were carefully mounted and supported between two polystyrene rings. The region of interest (L3-L5) was identified by radiography. High-resolution scanning was performed using 50 keV and 198 µA, employing a 0.5 mm aluminum filter, sample rotation of 360°, image rotation steps of 0.1° (3600 images per segment), frame averaging of 3, exposure times of 1.45 s, using an effective pixel size of 2.5 µm. A small vertical random movement was used to reduce ring artifacts caused by detector inhomogeneities^1^. Under these imaging conditions, spine segments of the younger animals were scanned within a single field of view, whereas in older animals with larger spine segments, overlapping stacked sub-scan segments were required (Table 1). These settings resulted in scan durations of 7 hours per scan for the 1–3 day old samples and 14 hours per scan for the 7–14 day old samples. Thereafter, all samples were stored in 70% Ethanol at 4 °C until further histological embedding and conventional optical microscopy (Fig. 3I).

Reconstruction was performed using the manufacturer software (NRecon, Version 1.6.8.0, Bruker MicroCT, Kontich, Belgium) with a unified attenuation (output) range of 0.0–0.15, chosen to avoid saturation of higher densities. The data were corrected for possible misalignments of overlapping sub-scans, mild beam hardening correction (20%), and ring artifact reduction (12). The resulting images were stored as 8-bit PNG images^6^. Two phantoms with densities of 0.25 g/cc and 0.75 g/cc were scanned and reconstructed using the same setup and protocols.

Additional details (Fig. 2) are provided in our related work^1^.

## Data Records

The reconstructed micro-CT dataset^6^ consists of 19 lumbar spine image sets of female C57BL/6 mice up to 2 weeks after birth, as well as 2 hydroxyapatite phantoms for calibration of mineral density values. Each image set contains between 1000 and 2300 8-bit BNP images. The datasets are available on Figshare. See Table 1 for an overview of the data files and their formats.

## Technical Validation

While regular instrument maintenance and flat-filed correction of reference scan states are routine and normal on the instrument, they are no guarantee for the quality of the data. To confirm the accuracy of our 3D analysis and imaging protocols we compared and matched the resulting micro-CT data to standard non-decalcified histomorphometry. The samples were embedded in Technovit 9100 PMMA (Kulzer GmbH, Hanau, Germany) after the micro-CT scans, cut into 5 µm thick sections (Leica SM2500S microtome) along the longitudinal axis, and stained with von Kossa/Toluidine blue to distinguish mineralized material and soft tissue (Fig. 3)^1^.

The 3D data of the reconstructed micro-CT scans were matched with the stained 2D slices using the oblique slice function of the 3D visualization program Amira (Ver. 5.4.0, Thermo Fisher Scientific, Berlin, Germany) (Fig. 3II). Using a series of 3 obliquely-resliced micro-CT images, architectural details were identified and match in the high-resolution images and in the histological stained sections (Zeiss Axioskop 2, Carl Zeiss Microscopy GmbH, Germany). In addition to visual identification of identical geometries of the trabeculae, we quantified a noteworthy high correlation (average R^2^ = 0.97) between standard 2D histomorphometric measurements (total area, bone area, B.Ar./Tt.Ar., Tb.Th, Tb.N, Tb.Le) and virtual slices in the 3D datasets, using the BoneJ plugin in ImageJ/FIJI, to confirm the technical reliability and accuracy of our data in the region of interest L3-L5^1,10,11^. The corresponding correlation for the histomorphometric parameter were: Total area R^2^ = 0.9999, bone area R^2^ = 0.9998, B.Ar./Tt.Ar. R^2^ = 0.9098, Tb.Th R^2^ = 0.9283, Tb.N R^2^ = 0.9988, Tb.Le R^2^ = 0.9857^1^. The correlation between the histomorphometry measurements and the measurements obtained from the resliced micro-CT images at 1-, 7-, and 14 days of age was remarkably high, despite the elaborate processing required for histology.

We conclude that our imaging- and data-processing protocols are therefore robust enough to precisely and realistically document the mineralized material deposition process over the range of sample ages, sizes, and mineral contents in our experiment.

## Usage Notes

The datasets are publicly accessible on Figshare^6^. Users and researchers are encouraged to make use of the data as a reference for physiological, cancellous bone growth in the lumbar spine of normal mice. The reconstructed datasets were reoriented, cleared from soft tissue using ImageJ (Fiji), and were analyzed using BoneJ, an open-source ImageJ (FIJI) plugin^1,10,11^. The comparison of micro-CT and histology was achieved using the oblique slicing function in Amira (Ver. 5.4.0, Thermo Fisher Scientific, Berlin, Germany). If required for specific applications, images, as well as entire image stacks, can be converted into other formats including TIFF or JPEG using open-access, free programs such as ImageJ (FIJI)^10^.

## Data Availability

No custom code was used in the data acquisition of these datasets.
